# Differences in EAG Response and Behavioral Choices between Honey Bee and Bumble Bee to Tomato Flower Volatiles

**DOI:** 10.3390/insects13110987

**Published:** 2022-10-27

**Authors:** Jinjia Liu, Jiangchao Zhang, Jinshan Shen, Huiting Zhao, Weihua Ma, Yusuo Jiang

**Affiliations:** 1 College of Animal Science, Shanxi Agricultural University, Jinzhong 030801, China; 2 College of Horticulture, Shanxi Agricultural University, Taiyuan 030031, China; 3 College of Life Sciences, Shanxi Agricultural University, Jinzhong 030801, China

**Keywords:** *Apis mellifera*, *Bombus terrestris*, EAG, behavior, tomato flower volatiles

## Abstract

**Simple Summary:**

Tomatoes are a popular crop, and bumble bees and honey bees are its main pollinators. Floral scent usually plays an important role in mediating the foraging behavior of bees, and tomato flowers release special scents. Although it has been found that foraging experience in the tomato greenhouses helped bumble bees develop a strong preference for the scent, honey bees with foraging experience continued to show aversion to tomato floral scent. However, it is currently unknown as to how a single tomato volatile compound regulates the foraging behavior of bees. In the current study, we investigated the foraging behaviors of the widely used pollinator honey bee *Apis mellifera* and bumble bee *Bombus terrestris* on tomato flower volatile compounds in order to evaluate whether honey bees and bumble bees show different EAG responses to volatile compounds and how they might influence bee choice behavior. We found that honey bees had a weaker EAG response to the tested compounds compared with bumble bees and that they showed avoidance behavior to these compounds. We conclude that some compounds in tomato floral scents caused the low bias of honey bees to tomato flowers, which may be one driver as to why honey bees dislike tomato, which could be adjusted in order to improve the pollination service efficiency of bees for commercial crops.

**Abstract:**

Bumble bees and honey bees are of vital importance for tomato pollination, although honey bees are less attracted to tomato flowers than bumble bees. Little is known about how tomato flower volatile compounds influence the foraging behaviors of honey bees and bumble bees. In this study, compounds of tomato flower volatiles were detected by gas chromatography–mass spectrometry. Electroantennography (EAG) and a dynamic two-choice olfactometer were used, respectively, to compare the differences of antennal and behavioral responses between *Apis mellifera* and *Bombus terrestris* towards selected volatile compounds. A total of 46 compounds were detected from the tomato flower volatiles. Of the 16 compounds tested, *A. mellifera* showed strong antennal responses to 3 compounds (1-nonanal, (+)-dihydrocarvone, and toluene) when compared with a mineral oil control, and *B. terrestris* showed 7 pronounced EAG responses (1,3-xylene, (+)-dihydrocarvone, toluene, piperitone, eucarvone, 1-nonanal, and β-ocimene). Additionally, 1-nonanal and (+)-dihydrocarvone elicited significant avoidance behavior of *A. mellifera*, but not of *B. terrestris*. In conclusion, bumble bees are more sensitive to the compounds of tomato flower volatiles compared to honey bees, and honey bees showed aversion to some compounds of tomato flower volatiles. The findings indicated that compounds of flower volatiles significantly influenced bee foraging preference for tomato.

## 1. Introduction

Tomato (*Lycopersicum esculentum*) is one of the most consumed vegetables worldwide. It is found ubiquitously and there are more than 7500 varieties, with a global annual value of USD 10.8 billion [1]. Tomato flowers are self-compatible and may be wind pollinated when planted in an open field; however, in order to enhance yield and improve physicochemical properties of their fruit, cultivated tomato and different pollination methods have been extensively studied in many countries [1,2,3,4]. China is the largest tomato producer in the world. To improve tomato yield and fruit quality, protected cultivation has become an important cultivation system, accounting for 57.2% of total tomato production in China [5]. However, ideal fruit set and quality are difficult to achieve without artificial or insect pollination, particularly under protected cultivation conditions, where wind and wild insects are absent [6].

The structure of tomato flowers is unusual among flowering plants [7]. Tomato flowers do not produce nectar and instead rely on pollen to attract and reward floral visitors. When the apex of the anther cone moves outwards, the exposed stigma is able to contact both buzzing and non-buzzing bees, releasing pollen from their poricidal anthers [8]. Manual pollination for tomato is also common, and it can be performed by simulating a bee’s vibration on the anther using a vibrating wand, but it is expensive and can damage the flower, and it is also usually less efficient than pollination by bees [9,10,11,12].

Bees have been widely considered to be the best insects for pollination [13]. Bumble bees are more efficient and reliable pollinators of greenhouse crops. The best-known bumble bee species, *Bombus terrestris*, native to Eurasia, has been exported worldwide for tomato pollination [14]. They are being commercially used for cultivated tomato pollination in many countries because of their buzzing pollination behavior and efficiency at low temperatures and low levels of sunlight, which help in high-quality fruit production [2,6,15,16,17]. *Apis mellifera*, which is the most widely used pollinator bee species in the world, can also pollinate tomatoes and improve fruit quality [3,16,18,19]. Although honey bees were reported to seldom visit tomato flowers as they obtain neither flower reward nor have the ability for buzz pollination, few studies have sought to understand the factors that could explain why honey bees are not suitable pollinators for tomatoes.

In nature, floral traits, mainly scents, are usually considered to be associated with flower rewards for pollinators, so they are also thought be one of primary driven factors that influence the foraging decision of bees [20,21,22,23]. Bumble bees develop a preference for synthetic volatiles after they obtain rewards from artificial flowers containing these compounds [24]. The foraging preference of *Bombus impatiens* to *Mimulus guttatus* has been found to be driven by odor cues, which have innately attractiveness to the bee [25]. Moreover, D-limonene, β-myrcene, and (*E*)-β-ocimene of the *M. lewisii* flower scent is sufficient in eliciting differential visitation by bumble bees [26]. Honey bee responses to floral scent have been widely studied, and flower scents of many crops, such as apple, pear, and kiwifruit, can stimulate bee antennae, allowing them to specifically distinguish between different plants or judge flower rewards [20,27,28]. It has also been reported that volatile organic compounds from tomato plants can influence the foraging behavior of herbivore pests [29,30,31]. Bumble bees prefer to visit tomato flowers containing less β-phellandrene and (+)-2-carene, causing a negative relationship that has been found between the daily release dynamics of these two compounds and the daily foraging activity of bumble bees [32]. However, it is difficult to conclusively determine the repellent effects of these two compounds on bumble bee foraging behavior. Whether tomato floral scent influences the foraging behaviors of bees and is thus responsible for the different pollination efficiencies of these two bee species in tomato is still unknown.

Bees play an important role in the pollination of tomatoes, yet the relationship between foraging choices and olfactory cues is unclear because of the difference in foraging behavior between honey bees and bumble bees on tomato flowers. In this study, the compounds of tomato flower scents were identified, and EAG response and foraging choices of honey bee *A. mellifera* and bumble bee *B. terrestris* on selected compounds were studied. We determined the electrophysiology and behavior responses of *A. mellifera* and *B. terrestris* to tomato flower compounds and analyzed whether differences existed between the two bee species. The results of this study will help to provide insight into bee–tomato interactions and guide future efforts to support tomato pollination.

## 2. Materials and Methods

### 2.1. Flower Volatiles Collection and GC–MS

Fresh tomato flowers (“4805Dahong”) were placed in a 20 mL vial sealed with 3 mL saturated NaCl solution and were equilibrated at 80 °C for 30 min. We then inserted 100 µL polydimethylsiloxane (PDMS) fibers (Supelco, St. Louis, MO, USA) into the vial for a 30 min extraction period at 80 °C. Before the test, PDMS fiber with tomato flower volatiles was placed in the inlet of the tube column at 240 °C for a 5 min desorption period.

Gas chromatography–mass spectrometry (Agilent 6890N-5975B) was used for qualitative and quantitative analysis as the following program: GC-fitted column: HP-5MS (0.25 mm × 30 mm × 0.25 µm); inlet temperature: 240 °C; helium carrier gas: percentage purity ≥ 99.99%; flow rate: 1.0 mL/min. The oven program was started at 45 °C, maintained for 5 min, heated from 45 to 130 °C at 6 °C/min, then from 130 to 240 °C at 10 °C/min, and finally maintained at 240 °C for 8 min. The injection was splitless. The MS parameters were as follows: the ion source temperature: 230 °C; the interface temperature: 250 °C. The ionization mode was electron ion source (EI). Full scanning was conducted at a mass scan range from m/z 45 to 500. Volatile compounds retrieval and identification were conducted using NIST 14 libraries, and the relative content of each component was analyzed by area normalization.

### 2.2. Preparation of Standard Compounds

The compounds β-caryophyllene, terpinolene, γ-terpinene, β-ocimene, (−)-β-pinene, *p*-cymene, 1,3-xylene, toluene, 2,4-dimethyl styrene, piperitone, eucarvone, (+)-dihydrocarvone, linalool, 1-nonanal, and tetradecane were diluted in mineral oil to six different concentrations (10 μg/μL, 100 μg/μL, 200 μg/μL, 300 μg/μL, 400 μg/μL, 500 μg/μL) for EAG and Y-tube behavioral experiments. All compounds were obtained from commercial suppliers (Table 1). We did not use the full range of standard compounds because some were unavailable at the time of our study.

### 2.3. EAG Responses

The procedure used to prepare the bees for the electroantennographic recording is described in [33] and was adapted to the special requirements of *A. mellifera* and *B. terrestris*. Honey bee workers were caught at the hive door and starved for 24 h before the experiments, and bumble bees were bought from company (Woofuntech bio-control, Hebei, China) a few days before the experiments. A bee was placed under an asana microscope, and one antenna was cut from the base using a scalpel blade. Then, the base had a reference glass electrode filled with Ringer’s solution in contact with an Ag/AgCl wire inserted into it. Following this, we used iris scissors to cut a small opening at the top of the antenna, exposing the internal tissue. Into this incision, we inserted a recording glass electrode also filled with Ringer’s solution.

The EAG responses were detected through a combi-probe (INR-II; Syntech, the Netherlands). The DC potential was recorded (Universal AC/DC probe), processed, and analyzed using EAG 2000 software (Syntech, Hilversum, the Netherlands). The purified air and testing compounds were provided by an air stimulus controller (CS55; Syntech, Hilversum, the Netherlands), with an 18 L/h constant air flow passed over. The testing antennae were adjusted to the center of the open end of the mental tube so that the air carried with compounds passed over was uniform. Each compound concentration was tested in a 1 min interval, and the pulse duration was 1 s. The time interval between each volatile compound was 2 min. All compounds were tested from low concentration to high concentration in order to avoid olfactory adaptation. A standard control mineral oil stimulation was performed at the beginning and the end of each recording as the blank control of the antennal responses. For each compound, EAG responses of at least four individual antennae of each bee species were recorded.

### 2.4. Y-Tube Olfactometer Test

Y-tube olfactometers (stem 25 cm, arms 18 cm, at an angle of 45°, internal diameter of 3.0 cm) were used for behavior choice tests to investigate the behavioral responses of bees to compounds. The test was performed in an odor-free room that was lit with a red LED bulb and maintained at around 25 °C. The olfactometer arms were connected to two glass gas desiccators, separately. Ten microliters of each compound was applied to a 3 × 1.5 cm^2^ filter paper strip that was immediately put into a glass gas desiccator. Each individual bee was supposed to make a choice between 10 µL of flower volatile compounds and 10 µL of mineral oil control. The compounds were evaporated for 30 s before air was passed from both arms to the stem; the air was cleaned by an activated charcoal filter and distilled water. The airflow pumped through each of the olfactometer arms was 500 mL/min. Bees were randomly collected from the entrances of the colonies and were observed independently for 5 min in the Y-olfactometer. For every five bees tested, the positions of the arms containing the control and treatment compounds were reversed, and a clean Y-tube was replaced for every 10 individuals tested. Bees who moved toward one of the compounds and stayed there for at least 5 s or moved two-thirds the length of a lateral arm were recorded as making successful choices. Each compound was tested with 30 *A. mellifera* individuals and 30 *B. terrestris* individuals separately, and then we analyzed the difference of choices.

### 2.5. Statistical Analyses

To analyze the content of volatiles, the peak area was proportionate to the quantity of total peak areas of an individual component. The EAG responses relative to the control (mineral oil) at different concentrations of each compound were presented as a percentage. Due to individual differences between bees, the data did not satisfy the normal distribution, and therefore non-parametric tests were used. Absolute mean EAG responses to the applied concentrations of the same odor compound were compared by one-way Kruskal–Wallis test. The preferences of the honey bees and bumble bees in the Y-tube olfactometer were analyzed with a chi-squared test (χ^2^) to explore whether significant differences existed in bees species, and data were transformed to percentages.

## 3. Results

### 3.1. GC–MS of Tomato Flower Volatiles

A total of 46 compounds were detected by GC–MS in tomato flower scents (Table 2). These components included a variety of olefins, as well as some ethers, alcohols, furans, aldehydes, and alkanes. Among these compounds, *p*-cymene and β-caryophyllene were the two most abundant compounds, with contents of 23.19% and 23.16%, respectively. In addition, the content of other compounds varied greatly, ranging from 0.04% to 11.74%.

### 3.2. EAG Response of Bees to Different Compounds

**Figure 1:**
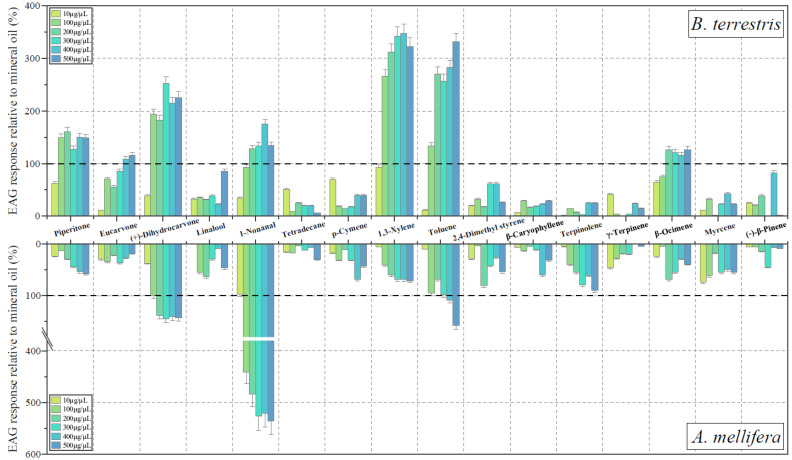
Normalized EAG responses of bees to volatile compounds. (**top**) Normalized EAG response relative to mineral oil of *B. terrestris*; (**bottom**) normalized EAG response relative to mineral oil of *A. mellifera*. The bolded black dotted line represents the response of the control mineral oil (100%).

The normalized EAG responses of *A. mellifera* and *B. terrestris* to *p*-cymene, 1,3-xylene, toluene, 2,4-dimethyl styrene, piperitone, eucarvone, (+)-dihydrocarvone, linalool, 1-nonanal, tetradecane, β-caryophyllene, terpinolene, γ-terpinene, myrcene, β-ocimene, and (−)-β-pinene at six concentrations (10, 100, 200, 300, 400, and 500 μg/μL diluted in mineral oil) are shown in Figure 1.

Antennal sensitivity to the different compounds was not consistent across species. Of these, antennal responses of *A. mellifera* to toluene, (+)-dihydrocarvone, and 1-nonanal were greater than to mineral oil, at least at the highest concentration of 100 μg/μL. The response to (+)-dihydrocarvone was not concentration dependent. In *B. terrestris*, responses to toluene, (+)-dihydrocarvone, and 1-nonanal were significantly stronger than responses to mineral oil, and several others such as 1,3-xylene, piperitone, eucarvone, and β-ocimene were also stronger than mineral oil. The eucarvone did not elicit the antennal response at lower doses (10, 100, 200, 300 μg/μL). Conversely, the compounds toluene, 1,3-xylene, eucarvone, and β-ocimene elicited obvious concentration-dependent EAG responses. *A. mellifera* and *B. terrestris* showed significant species-specific differences between the amplitudes of their EAG response, with *B. terrestris* having a greater response to these compounds than *A. mellifera*.

The absolute mean EAG responses of bee species that had significant differences from volatile compounds at the different concentrations are shown in Table 3. For *B. terrestris*, there were three compounds that had significant differences among concentrations. The antennae responses to *p*-cymene (*p* = 0.006, Kruskal–Wallis test), toluene (*p* = 0.047, Kruskal–Wallis test), and 2,4-dimethyl styrene (*p* = 0.041, Kruskal–Wallis test) were positively correlated with concentrations. Moreover, 1,3-xylene at a concentration of 400 μg/μL, (+)-dihydrocarvone at a concentration of 300, and toluene at a concentration of 500 μg/μL were the top three highest EAG responses of *B. terrestris*.

*Apis mellifera* showed no significant difference from the tested compounds in terms of EAG response, except toluene (*p* = 0.015, Kruskal–Wallis test). The top three highest EAG responses of *A. mellifera* were to 1-nonanal at a concentration of 500 μg/μL (*p* > 0.05, Kruskal–Wallis test), (+)-dihydrocarvone at a concentration of 300 μg/μL (*p* > 0.05, Kruskal–Wallis test), and toluene at a concentration of 500 μg/μL.

### 3.3. Y-Tube Tests of Bee Choice to Compounds

Volatile compounds showed significantly different influence on the choice behavior of honey bees and bumble bees. Overall, *A. mellifera* showed significant avoidance responses to these three compounds, while *B. terrestris* showed being weakly attracted or remaining neutral.

**Figure 2:**
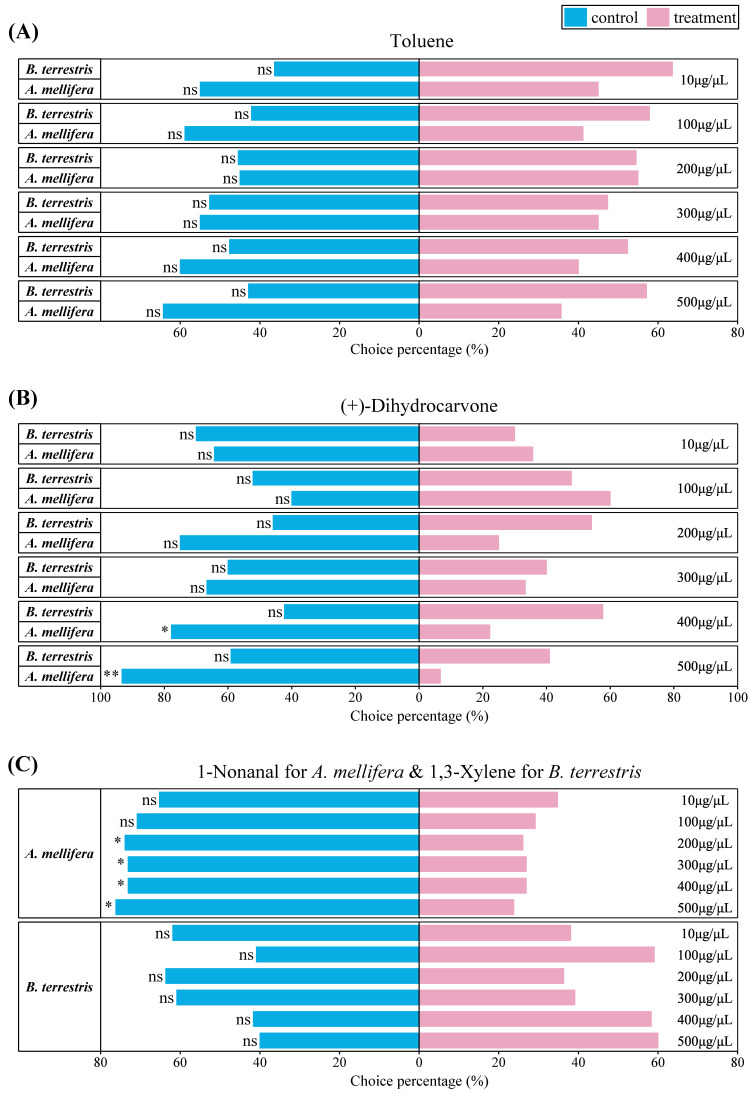
Y-tube behavior choices of bees towards different compounds at different concentrations. (**A**) The choice percentage of *B. terrestris* and *A. mellifera* to toluene (ns: *p* > 0.05). (**B**) The choice percentage of *B. terrestris* and *A. mellifera* to (+)-dihydrocarvone (ns: *p* > 0.05; * 0.01 < *p* < 0.05, and ** 0.001 < *p* < 0.01). (**C**) The choice percentage of *A. mellifera* to 1-nonanal and *B. terrestris* to 1,3-xylene (ns: *p* > 0.05; * 0.01 < *p* < 0.05).

For toluene, neither *B. terrestris* nor *A. mellifera* showed significant behavioral responses of avoidance or attraction (Figure 2A). At five concentrations (10, 100, 200, 400, and 500 μg/μL), *B. terrestris* showed a selection rate greater than 50%, but there were no statistically significant differences (*p* > 0.05, chi-squared tests). In contrast, the choice percentages of *A. mellifera* tending towards mineral oil control were higher than towards toluene, except at the concentration of 200 μg/μL (55%), although there were no significant differences in behavioral responses at all six concentrations (*p* > 0.05, chi-squared tests).

For (+)-dihydrocarvone, the behavioral responses of *B. terrestris* and A. *mellifera* were distinctly different (Figure 2B). The choice percentages of *B. terrestris* still did not have a significant difference (*p* > 0.05, chi-squared tests), and there were more *B. terrestris* individuals that tended towards the mineral oil control at concentrations of 10 and 500 μg/μL, while more individuals chose (+)-dihydrocarvone at the other three concentrations. *A. mellifera* exhibited an avoidance response to (+)-dihydrocarvone—the avoidance rate was as high as 99.33% at 500 μg/μL (χ^2^ = 11.267, df = 1, *p* = 0.001) and 77.78% at 400 μg/μL (χ^2^ = 5.556, df = 1, *p* = 0.023), but more bees were attracted to (+)-dihydrocarvone at the concentration of 100 μg/μL (χ^2^ = 0.80, df = 1, *p* = 0.24).

For 1,3-xylene, *B. terrestris* showed different behavioral responses at different concentrations (Figure 2C, bottom). At concentrations of 10, 200, and 300 μg/μL, more *B. terrestris* individuals tended towards the mineral oil control (61.9%, 63.64%, and 60.87, respectively); at the other three concentrations, *B. terrestris* showed an attractive response with choice percentages towards 1,3-xylene being 59.1% for 100 μg/μL, 58.3% for 400 μg/μL, and 60.0% for 500 μg/μL. However, there were no significant statistical differences in all six concentrations (*p* > 0.05, chi-squared tests).

For 1-nonanal, *A. mellifera* showed an avoidant behavior from the 10 μg/μL concentration to 500 μg/μL concentration, being negatively correlated with concentration and the repellent rate (Figure 2C, up). There were significant differences in repellent rates of 1-nonanal from 200 μg/μL concentration to 500 μg/μL concentration: 73.91% for 200 (χ^2^ = 5.261, df = 1, *p* = 0.024), 73.08% for 300 (χ^2^ = 5.538, df = 1, *p* = 0.02), 73.08% for 400 (χ^2^ = 5.538, df = 1, *p* = 0.02), and 76.19% for 500 (χ^2^ = 5.762, df = 1, *p* = 0.019).

## 4. Discussion

Floral scents are secondary metabolites of plants, functioning to attract insect pollinators and mediate the foraging behavior of bees [34]. Both bumble bees and honey bees have been reported to be important pollinators for tomatoes, and tomato flowers release special scents. The findings of this study revealed that honey bees and bumble bees had different EAG responses to tomato flower volatiles, and the two bee species showed different behavioral choices to the same compound. The foraging choices of honey bees and bumble bees may be different when pollinating in cultivated tomatoes. Therefore, using bumble bees for pollination in cultivated tomatoes is more efficient, but some effective measures can also be taken to improve the attractiveness of tomato flowers to honey bees. This study provides evidence for honey bees as unreliable pollinators for tomato pollination, which will help to further understand the plant−insect interrelationship.

Plant floral scents play crucial roles in mediating the foraging behavior of insect pollinators [35]. Floral volatiles are frequently inconsistent among times, locations, collection methods, and even duplicate samples collected at the same time. *p*-Cymene, terpinolene, and β-caryophyllene were the top three compounds with the highest content detected from our tomato flower samples, but neither bee species produced an antenna response to them, neither at high nor low concentrations. Other studies found that the volatiles collected from tomato flowers were dominated by α-pinene, *p*-cymene, 2-carene, and β-phellandrene, and *Bombus impatiens* preferentially foraged on flowers that released less β-phellandrene and (+)-2-carene relative to flowers that released higher amounts of these volatiles [32]. In addition, there are several compounds, such as linalool, that do not cause antennae responses of bees, although (8*S*,9*R*)-(*E*)-caryophyllene, *p*-cymene, α-terpinene, and linalool have been proven to be electrophysiologically attractive for bees or *Aphidius ervi* [28,29,36,37]. It can be seen that the relationship between the concentration of flower volatiles and bees is complex. Plant defense against herbivores may show a trade-off with pollinator attraction [38]. Therefore, the trade-off between herbivore defense and pollinator attraction in tomato plants requires further investigation.

Plant odors can attract and repel different bee pollinators [39]. Bees are able to perceive volatile molecules from flowers and respond differently to volatile compounds. The EAG response results in our study indicated that honey bees are less sensitive to tomato flower scents compared to bumble bees. Of all tested compounds, only 1-nonanal, (+)-dihydrocarvone, and toluene elicited strong antennal responses of *A. mellifera* when EAG responses were normalized to mineral oil. Moreover, these three compounds that caused *A. mellifera* antennae responses were all repellent to bees, especially 1-nonanal. Similarly, it was also confirmed that 1-nonanal is a repellent for both *A. cerana* and *A. mellifera* [40]. Dihydrocarvone is a proven repellent, with contact and fumigant toxicity to *Rhyzopertha dominica*, *Sitophilus oryzae*, *Tribolium castaneum* adults, and *T.castaneum* larvae at varying degrees [41]. Constituents from many spices and herbs are known to have insecticidal activities including fumigant and topical toxicity as well as antifeedant or repellent effects [42]. This may be a defense mechanism for plants, using special compounds to avoid predators or excessive ineffective pollination. It has been reported that volatile organic compounds from tomato plants can influence the foraging behavior of herbivore pests [29,31]. Honey bee antennae responses to very few compounds may explain their negative foraging behaviors of tomato flowers.

Volatile pheromones and plant aromas are essential sensory criteria for bumble bees in order to ensure the survival of the species. *Bombus terrestris* displayed pronounced antennal responses to seven compounds, showing they have greater discernment than honey bees. It has been widely known that bumble bees are effective bee pollinators of tomato as they are capable of buzz pollination to vibrate tomato anthers in order to release pollen grains, and only one visit is sufficient for full tomato pollination [43]. However, the innate responses of insect pollinators to plant floral scent are not always consistent with their foraging behaviors on plants [18]. These three compounds (1,3-xylene, (+)-dihydrocarvone, and toluene) also do not have an extremely behavioral attraction when compared to the apparent antenna potential responses in our study. Additionally, tomato flowers produce certain chemicals (β-phellandrene and 2-carene) that reduce the visitation frequency of *B. impatiens* to the flowers, thus impeding bee pollination [18]. The amount of these chemicals can be altered by different cultivation practices: vegetative plants produced less β-phellandrene and 2-carene and received more visits than generative plants [44]. Generally, pollinator behavioral responses to floral scent are dependent on both innate bias and learned experience, as some have shown modest aversion to tomato floral scent [18].

## 5. Conclusions

Honey bees exhibited aversion to compounds of tomato flower volatiles while bumble bees did not. In our study, honey bee antennae responded to only three compounds, fewer than the seven compounds to which bumble bee antennae responded. All these compounds were repellent to honey bees in behavior choice tests. These information may explain why tomatoes are not attractive to bees. However, in the present study, not all detected volatile compounds were obtained and used to test honey bees and bumble bees. More tests of other compounds are needed to fully explain the interaction of tomato and pollination bees. Further study could focus on the application of volatile compounds in pollination management to improve the honey bee pollination efficiency of tomato.

## Figures and Tables

**Table 1:** Volatile compounds used as olfactory stimuli in the EAG and the Y-tube experiments tested with pollinators *A. mellifera* and *B. terrestris*.

No.	Compounds	CAS Number	Company	Purity
1	β-Caryophyllene	87-44-5	Macklin ^1^	>80%
2	Terpinolene	586-62-9	Aladdin ^2^	85%
3	γ-Terpinene	99-85-4	Aladdin	>95%
4	β-Ocimene	13877-91-3	Aladdin	>90%
5	Myrcene	123-35-3	Aladdin	≥90.0%
6	(−)-β-Pinene	18172-67-3	Aladdin	98%
7	*p*-Cymene	99-87-6	Aladdin	≥99.5%
8	1,3-Xylene	108-38-3	Aladdin	>99.0%
9	Toluene	108-88-3	Sigma-Aldrich ^3^	99.80%
10	2,4-Dimethyl styrene	1195-32-0	Aladdin	>95%
11	Piperitone	89-81-6	Aladdin	>94.0%
12	Eucarvone	503-93-5	Macklin	≥96%
13	(+)-Dihydrocarvone	7764-50-3	Aladdin	98%
14	Linalool	78-70-6	Aladdin	98%
15	1-Nonanal	124-19-6	Aladdin	96%
16	Tetradecane	629-59-4	Aladdin	>99%
17	Mineral oil	8042-47-5	Aladdin	99%

^1^ Macklin: Shanghai, China; ^2^ Aladdin: Shanghai, China; ^3^ Sigma-Aldrich: Steinheim, Germany.

**Table 2:** Number and relative peak area (% of single compounds) of compounds detected in flower volatile samples of tomato.

No.	CAS	Compounds	Relative Content (%)
1	115-10-6	Dimethyl ether	0.057
2	645-88-5	O-(Carboxymethyl)hydroxylamine	0.058
3	87980-11-8	3-Amino-2,3-dihydrobenzoic acid	0.946
**4**	**108-88-3**	**Toluene**	**0.389**
**5**	**108-38-3**	**1,3-Xylene**	**0.079**
6	3479-89-8	1,3,5-Cycloheptatriene, 3,7,7-trimethyl-	0.537
**7**	**99-87-6**	***p*** **-Cymene**	**23.185**
8	460-01-5	2,6-Dimethyl-1,3,5,7-octatetraene, E,E-	0.119
**9**	**18172-67-3**	(**−**)**-β-Pinene**	**0.309**
10	127-91-3	β-Pinene	3.23
**11**	**123-35-3**	**Myrcene**	**3.23**
12	28634-89-1	Bicyclo [3.1.0]hex-2-ene, 4-methyl-1-(1-methylethyl)-	0.083
13	527-84-4	ο-Cymene	0.891
14	3779-61-1	(*E*)-β-Ocimene	0.672
**15**	**13877-91-3**	**β-Ocimene**	**4.488**
**16**	**99-85-4**	**γ-Terpinene**	**4.605**
**17**	**1195-32-0**	**2,4-Dimethyl styrene**	**0.135**
**18**	**586-62-9**	**Terpinolene**	**11.736**
**19**	**78-70-6**	**Linalool**	**0.145**
**20**	**124-19-6**	**1-Nonanal**	**0.1**
21	18368-95-1	1,3,8-ρ-Menthatriene	0.405
22	38667-10-6	3,3,5,5-Tetramethylcyclopentene	2.764
23	21391-98-0	1-Cyclohexene-1-carboxaldehyde,4-(1-methylethyl)-	1.131
24	70786-44-6	3,6-Dimethyl-2,3,3a,4,5,7a-hexahydro-benzofuran	1.304
**25**	**7764-50-3**	(**+**)**-Dihydrocarvone**	**0.143**
26	1197-06-4	2-Cyclohexen-1-ol,2-methyl-5-(1-methylethenyl)-, (1R,5R)-rel-	0.044
**27**	**503-93-5**	**Eucarvone**	**0.085**
**28**	**89-81-6**	**Piperitone**	**0.395**
29	20307-84-0	(+/−)-δ-Elemene	4.403
30	17699-14-8	(−)-α-Cubebene	0.051
31	469-92-1	(−)-Clovene	0.049
32	3856-25-5	α-Copaene	0.51
33	515-13-9	β-Elemene	0.867
**34**	**629-59-4**	**Tetradecane**	**0.166**
35	118-65-0	Isocaryophyllene	0.137
**36**	**87-44-5**	**β-Caryophyllene**	**23.164**
37	136296-38-3	10,10-Dimethyl-2,6-dimethylenebicyclo [7.2.0]undecane	0.302
38	29873-99-2	γ-Elemene	0.136
39	6753-98-6	α-Caryophyllene	5.545
40	483-77-2	(−)-Calamenene	0.176
41	95910-36-4	(−)-Isoledene	1.074
42	523-47-7	β-Cadinene	1.435
43	483-76-1	Δ-Cadinene	0.224
44	6813-21-4	Selina-3,7(11)-diene	0.16
45	1139-30-6	Caryophyllene oxide	0.132
46	77171-55-2	Spathulenol	0.205

The bold font represents the compounds we purchased for the use of EAG and Y-tube test.

**Table 3:** Absolute mean EAG response with significant differences of *A. mellifera* and *B. terrestris* to different concentrations of the same compound.

Absolute Mean EAG Response to Different Compounds (Diluted in Mineral Oil)												
CONC (μg/μL)	*p*-Cymene		1,3-Xylene		Toluene		2,4-Dimethyl Styrene		(+)-Dihydrocarvone		1-Nonanal	
	*B. terrestris*	*A. mellifera*	*B. terrestris*	*A. mellifera*	*B. terrestris*	*A. mellifera*	*B. terrestris*	*A. mellifera*	*B. terrestris*	*A. mellifera*	*B. terrestris*	*A. mellifera*
10	0.61 ± 0.24 b	0.11 ± 0.13 a	0.70 ± 0.61 a	0.13 ± 0.26 a	0.04 ± 0.06 b	0.07 ± 0.11 b	0.10 ± 0.09 b	0.13 ± 0.04 a	0.27 ± 0.10 a	0.45 ± 0.41 a	0.22 ± 0.34 a	0.86 ± 0.63 a
100	0.16 ± 0.18 b	0.24 ± 0.15 a	1.99 ± 1.43 a	1.37 ± 0.66 a	0.41 ± 0.14 a	1.35 ± 0.45 a	0.17 ± 0.10 ab	0.12 ± 0.14 a	1.38 ± 0.16 a	1.06 ± 0.65 a	0.61 ± 0.70 a	4.12 ± 1.92 a
200	0.12 ± 0.25 b	0.07 ± 0.15 a	2.34 ± 1.66 a	1.31 ± 0.63 a	0.84 ± 0.23 a	1.25 ± 0.40 a	0.61 ± 0.24 a	0.37 ± 0.20 a	1.31 ± 0.38 a	1.16 ± 0.64 a	0.84 ± 0.72 a	4.32 ± 2.02 a
300	0.73 ± 0.06 a	0.19 ± 0.14 a	2.57 ± 1.84 a	1.52 ± 0.64 a	0.80 ± 0.18 a	1.47 ± 0.37 a	0.84 ± 0.27 a	0.20 ± 0.13 a	**1.80 ± 0.46 a**	**1.75 ± 0.72 a**	0.87 ± 0.93 a	4.70 ± 2.17 a
400	0.54 ± 0.11 a	0.42 ± 0.20 a	**2.61 ± 2.00 a**	1.52 ± 0.68 a	0.87 ± 0.30 a	1.55 ± 0.33 a	0.84 ± 0.36 a	0.12 ± 0.10 a	1.54 ± 0.56 a	1.69 ± 0.78 a	1.14 ± 0.87 a	4.65 ± 2.13 a
500	0.53 ± 0.14 a	0.27 ± 0.09 a	2.42 ± 1.85 a	1.57 ± 0.79 a	**1.03 ± 0.36 a**	**1.91 ± 0.34 a**	0.65 ± 0.22 a	0.25 ± 0.20 a	1.61 ± 0.48 a	1.72 ± 0.81 a	0.88 ± 0.94 a	**4.78 ± 2.24 a**

The bold font represents the top three antennae responses of bees to compounds. Different letters indicating the significant difference among different concentrations of same compounds (*p* < 0.05, one-way ANOVA followed by Kruskal–Wallis test). Values are means (± standard error, *n* = 4 for *B. terrestris*, *n* = 6 for *A. mellifera*).

## Data Availability

The data that support the findings of this study are available from the corresponding author upon reasonable request.
